# How psychology might alleviate violence in queues: Perceived future wait and perceived load moderate violence against service providers

**DOI:** 10.1371/journal.pone.0218184

**Published:** 2019-06-24

**Authors:** Dorit Efrat-Treister, Arik Cheshin, Dana Harari, Anat Rafaeli, Shira Agasi, Hadar Moriah, Hanna Admi

**Affiliations:** 1 Department of Management, Ben-Gurion University of the Negev, Beersheba, Israel; 2 Department of Human Services, University of Haifa, Haifa, Israel; 3 Scheller College of Business, Georgia Institute of Technology, Atlanta, Georgia, United States of America; 4 Faculty of Industrial Engineering and Management, Technion-Israel Institute of Technology, Haifa, Israel; 5 Department of Nursing, The Max Stern Yezreel Valley College, Yezreel Valley, Israel; Monash University, AUSTRALIA

## Abstract

**Introduction:**

Queues are inherent to service encounters, as it is not always possible to provide service to all clients at the exact moment they request service. Queues involve waiting for a service in a specific place that might also be crowded, they obstruct the client's’ goal of receiving service, and at times lead clients to mistreat service providers and in extreme cases even attack them violently. We show, in a hospital setting, that perceived predicted future wait and load can buffer the causes of violence towards service staff.

**Methods:**

We combine objective data on crowdedness, reports of violence, and durations of time people waited, with psychological measures of perceived load and perceived future wait, collected from 226 people in the Emergency Department (ED) of a large hospital. Visitors to the ED were recruited as they waited for service. They indicated their perceived load in the ED and their perceived remaining wait for service. This data was then triangulated with objective operational data regarding the actual number of people waiting for service (i.e., crowdedness) and objective data regarding staff calls to security to stop violent accounts.

**Results:**

We find that with increased crowdedness, there are more calls to security reporting violence. However, this relationship is moderated by two factors: when people perceive the future wait to be short and when they perceive the load on the system to be high. Moreover, a three-way interaction shows that crowdedness is associated with more incidents of violence, however high perceived load and low perceived future wait are associated with fewer violent incidents.

**Conclusions:**

This paper demonstrates the relationship between crowded queues and violence towards service staff, and suggests two psychological mechanisms for buffering such violence: reducing perceived future wait and elevating perceived load.

## Introduction

Queues and wait are an inevitable part of service delivery [1]. Queues are notoriously stressful and frustrating, especially if they are long and slow moving. Long queues obstruct goal achievement [2,3,4] and create an experience of crowdedness, that can elevate client aggression towards service providers [5], and in extreme cases even violently [6,7,8]. Efforts to reduce such aggression and violence include increasing the number of available service staff (which should objectively make the queue move faster and reduce crowdedness) and enlarging the waiting area (which should make the queue appear shorter and reduce perceived crowdedness [9]). These options are, however, expensive [10] and often unavailable in many service contexts such as hospital Emergency Departments (EDs), the setting examined in the current paper. Hospital EDs, suffer from long wait times and crowdedness, both of which have a major influence on client violence [11].

Client violence towards service providers is alarmingly frequent worldwide [12,13,14], especially in hospital EDs, where its frequency is extremely high [11]. Indeed, in the US, 82% of service providers have reported being witness to or the target of violence in any given year [15]. Unfortunately, it seems that client violence continues to grow, in the health sector the reports of violence against nurses in the US have doubled in recent years [16].

Client violence against staff has substantial costs and implications for service management. The direct financial cost of violence in hospitals is estimated as $100,000 per case in the USA [10], and the UK National Health Service estimates the annual cost of violence at £69 million in the UK alone [6]. Violence leads to impaired staff performance [17,18], increases error frequency [19], and elevates levels of employee exhaustion, burnout, and turnover [20,21,22,23]. Indeed, extreme forms of aggression, i.e. violence, have caused physical injuries [14,16,24], and even murder [25]. Despite its increasing prevalence and serious implications, queue-related violence is under-studied and poorly understood [26,27,28,29].

To begin to address this crucial question, both theoretically and practically, we aim to integrate available research on the relationship between operational aspects of queues [30] and psychological aspects of waiting in queues [31,32,33,34]. By so doing, we strive to better understand how an extreme form of staff mistreatment and violent attacks against the staff might be modified [34,35]. We draw on the psychological model of stress and coping [4,36] to suggest that psychological perceptions about future wait time and the perception of load relate to violence towards service providers, above and beyond the operational aspects of queues. Our research focuses on the service field, specifically the health care in hospital EDs, where wait times and crowdedness have a major influence on patient violence, and where the frequency and costs of violence are perhaps the most severe [11].

Our paper makes two main contributions. First, from a conceptual standpoint, we combine psychological (subjective) and operational data (objective) to examine their combined effects on violence in queues: (i) operational aspects of the queue (specifically, wait time and crowdedness); (ii) psychological perceptions of the queue (specifically perceived future wait and perceived load); and (iii) calls to security to report violent attacks against the staff. This integration of operational and psychological measures advances our understanding of queues in service settings [33] and, more specifically, violence against staff. Second, we probe the effects of two psychological factors–perceived future wait time and perceived load–on violence in service settings. Specifically, our analysis examines whether clients’ *perceptions* of operational factors (rather than the operational, objective factors themselves) are related to levels of violence against service staff members.

## Literature review

Research on violence and aggression in field settings is lacking [37,38,39]. Aggression is defined as “intentional behavior directed at doing harm to a living being whether harm results or not, that can be physical or verbal, active or passive, and can be directly or indirectly focused on the victim(s)” [7]. Violence is the extreme end of aggression, and is our focus in this paper, defined as “any aggressive act that has as its goal extreme physical harm” [40] p246. We draw from literature on aggression, which is far more developed, to predict *when* violence will occur, since violence typically occurs as an escalation of aggression. The prevailing general aggression model [40] describes the likelihood of an individual behaving aggressively towards another person and suggests that there are proximate processes and distal causes and processes that lead to different levels of aggression. Our current paper examines proximate processes that can explain individual episodes of aggression, concentrating specifically on the situational factors that cause extreme aggression, and thus violence. The queue context is characterized by high stress, a sense of rejection, frustration, anonymity, bad moods, noise, heat, fear, identity threat, and a sense of injustice, all of which may trigger aggression that might escalate into violence [39,40,41,42,43,44].

Using a psychological framework, we treat operational aspects of queues (crowdedness and wait time) as stressors [30,45], or operational constraints that hinder desired goals [46]. We investigate how these stressors lead to emotions that may manifest behaviorally in the form of violence. Against this backdrop, we propose two psychological aspects of queues–predicted future wait time and perceived load–that may moderate the effects of queueing stressors. We investigate the separate and combined effects of these factors on aggression towards service staff.

## Crowdedness as a stressor

Crowdedness is defined objectively as the density of people in a specific space [47]. From an operations perspective, crowdedness is the extent to which demand for a service exceeds the ability to deliver that service in a timely fashion [48]. Simply put, crowdedness reflects the number of people competing for the system’s attention [49,50]. Crowdedness also has a psychological quality. Research in the social sciences shows that crowdedness, oftentimes referred to as load, violates people’s sense of personal space and leads to feelings of helplessness [51,52,53,54,55]. These feelings increase arousal and a tendency to blame others for adverse situations [56]. Moreover, increased temperature, a characteristic of crowded environments, is an antecedent of aggression and violence [57]. Altogether this means crowdedness increases stress [58], and as such can beget destructive behaviors such as aggression and violence [40,59,60,61]. Thus, our first hypothesis connects crowdedness to violence, predicting that a more crowded waiting area will lead to more reports of violence towards service staff.

## Wait time as a stressor

A second operational aspect of queues that acts as a stressor is the amount of time spent waiting [33]. Time is a resource with significant monetary and personal value [62,63,64,65], and spending this valuable resource waiting for service (or in common parlance, “wasting time”) is frustrating [66]. This frustration increases the likelihood of aggression and violence [2,3,4,60]

Time spent waiting has an objective value, which we refer to as *past wait time*, and define as the amount of time one has already waited. However, time also has a psychological value, that relies on perceptions that affect behavior separate from the actual time spent waiting [67,68,69, 70,71,72,73]. There are psychological perceptions of the time a person has *already spent waiting*, or of the time one must still wait. We focus on the latter, which we label “*perceived future wait*” [74]. We propose that perceived future wait, is a critical key to buffering violence against service staff in queues, as when it is short, it induces hope [75].

## Perceived future wait as a buffer of violence

A particularly stressful aspect of queuing is the uncertainty regarding the length of time remaining before the person waiting reaches the head of the queue and is served [76]. We suggest that even under crowded conditions, a short perceived future wait time can offer hope, and this effect is strong enough to buffer the harmful psychological effects of crowdedness (the violations of personal space, frustration, sense of helplessness, and sensory load). Hope, or “belief in the possibility of a favorable outcome” [77], is linked to future-oriented and positive thinking [78]. Hope in this respect is situational, and represents people’s speculations regarding how much longer they expect to remain waiting. As such, it is a source of motivation [79] and a vital coping resource that can help buffer the stress of waiting [77]. According to this reasoning, a short perceived future wait reduces the stress and frustration of waiting as well as the tendency to act violently. We predict the more crowded the queue, the more distressing the situation, and the more important the length of perceived future wait in buffering the effects of crowdedness on violence. Therefore, our second hypothesis posits that perceived future wait buffers the relationship between crowdedness and violence towards service staff, such that the shorter the perceived future wait the weaker the relationship between crowdedness and violence.

## Perceived load as a buffer of violence

A key to understanding the dynamics of a given queue is how many people are present, which is defined as the *system load* [80]. Aside from the actual system load, people attribute meanings to the number of people present, which we define as people's *perceived load*. We posit that perceptions of load are critical in shaping the behavior of people waiting, *because these perceptions help people understand why they are waiting*. These perceptions in turn influence behavior, through a process known as “sensemaking” [81,82], which describes how people experience and react to a given situation [83]. A large number of people waiting in a queue leads the people waiting to perceive the system as loaded [84] and enhances perceived wait durations [85,86,87]. Such perceptions of high workload lead people to adjust their expectations. Thus, our third hypothesis predicts that perceived load buffers the relationship between crowdedness and violence towards service staff, such that the higher the perceived load the weaker the relationship between crowdedness and violence.

## Overview of hypotheses

In sum, our first hypothesis (H1) is that crowdedness is associated with increased incidents of violence. In Hypotheses 2 and 3, we posit that perceived future wait (H2) and perceived load (H3) moderate the relationship between crowdedness and violence. The final leg of our argument integrates the two latter factors–perceived future wait and perceived load. We suggest that the combined effect of perceived future wait and perceived load synergistically buffers the effect of crowdedness on violent attacks against service staff. Specifically, we predict that the weakest relationship between objective crowdedness and violence occurs when the perceived future wait is lowest and the perceived load is highest. In other words, in our fourth and final hypothesis we propose a three-way interaction; crowdedness, perceived future wait, and perceived load have a combined three-way interactive relationship with violence towards service staff, such that crowdedness is associated with increased incidents of violence, however violence is lowest when perceived future wait is lowest and perceived load is highest.

**Fig 1:**
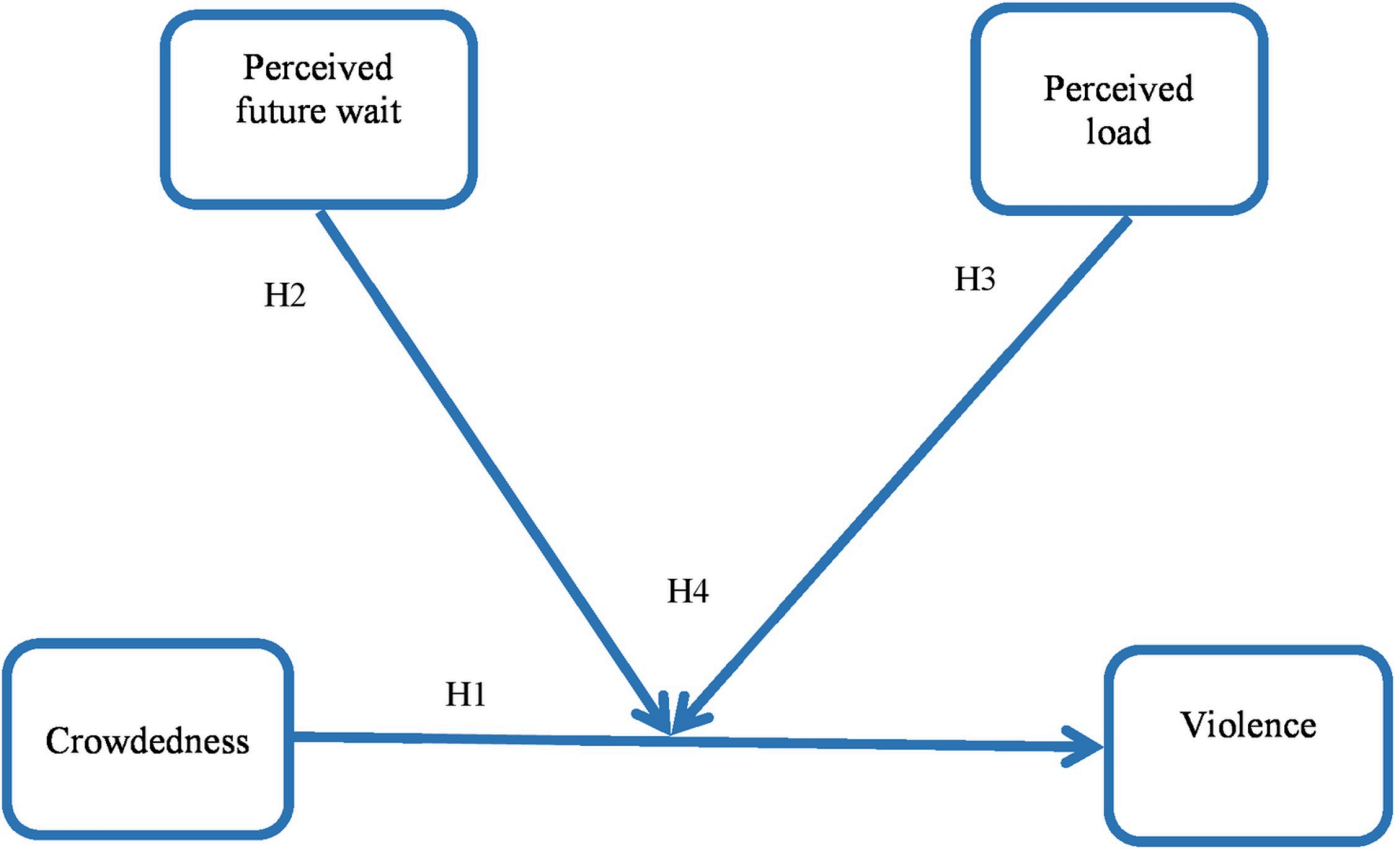
Research model.

Fig 1 visually summarizes our research model and four hypotheses.

## Materials and methods

### Setting

We tested our hypotheses using data we collected in the waiting area of the main Emergency Department (ED) of a large hospital over an eight-month period. This hospital is a public, university hospital, with over 1000 beds, having modern medical facilities and units, and is located in northern Israel. The hospital ED treats an average of 95,000 patients annually [28]. The hospital ED includes a “main ED”, and several other sections (e.g., “children’s ED”, “women’s ED” etc.); our study addressed only the main ED section. All patients in the main ED are not hospitalized and are physically capable of standing and moving around. Patients who are in beds are in a separate ED ward. The ED environment is a strategic context for testing our hypotheses, as it is characterized by long queues [29], is often crowded [30], and is the arena of frequent violence towards service staff [13,88].

### Procedure and participants

We triangulated three sources of data; hospital records on patient numbers in the ED, security records on the number of violent incidents reported, as well as survey responses from people waiting in the ED. Our data included objective field data (extracted from hospital records) and psychological data (collected through surveys of people waiting in the ED). The objective field data included *crowdedness* (number of patients in the ED at a given time), *past wait* (time a person had already waited, used as a control), and *violence reports* (number of emergency calls made to security by staff). The psychological (self-reported) data included *perceived future wait* and *perceived load*.

Surveys were collected by Research Assistants (RA), who randomly approached people waiting in the waiting area of the main ED asking them to respond to a short survey in exchange for a snack coupon. These approached individuals were both patients and patient escorts (e.g., family members, friends) There were no significant differences between responses of patients and escorts. Data were collected at multiple time points, between December 6th, 2009 and October 4th 2010 and included in total 19 different visits; RA’s visited the ED at randomly determined times which included all hours of a day and all weekdays. Data were not collected on the weekends as these times are characterized by lower staffing, different levels of crowdedness, and potentially different patient populations and atmosphere. Responses to the survey were anonymous, and respondents indicated the time they entered the ED (copied from their medical folders of the patients) and the time at which they responded to the survey, which allowed us to correspond the survey data with hospital records and security reports.

## Measures

### Objective data

#### Crowdedness

Crowdedness was defined as the number of patients in any part of the ED, including the waiting area and any of the treatment stations, as extracted from the hospital medical database. The number of patients at a given time (t_j_) is defined:

*Crowdedness* = *N patients entered ED until time t_j_*−*N patients left ED until time t_j_*.

(j stands for the specific patient. t_j_ stands for the time each patient completed the survey. This allows for a dynamic measure. Crowdedness is calculated as the number of patients who entered the ED until time t_j_.)

This dynamic measure of crowdedness was calculated when the participant was given the survey. To capture a respondent's actual experience of crowdedness, we averaged the ED crowdedness score over the 30 minutes before and after the time of completing the survey. This also accounted for the variability in the time it took participants to respond to the survey (4 to 15 minutes).

#### Past wait

Wait time was defined as the actual time (in hours and minutes) that passed since a survey respondent entered the ED and was calculated as follows:

$$Past\;wait\;\left(patient{\;}_{k}\right)\;=\;T\;patient_{k}\;responded\;to\;survey\;–\;T\;patient_{k}\;entered\;ED.$$

Past wait was used in the analysis as a control variable, following Carlson and Wu’s [89] work, as there is a theoretical basis for predicting the influence of past wait on perceived load.

#### Violence

All hospital rooms and facilities are equipped with an emergency button that can be used by staff to call a security guard when they encounter any form of aggression or threat that is directed towards themselves or others around them. All calls are recorded in a central security database from which we extracted data regarding reported incidents. Each call is recorded with a date and time stamp, which allowed us to link these data with the other measures. To test our hypotheses, we calculated the number of reports that occurred in the ED within 30 minutes before and after the time they filled in the survey (i.e., an hour around the survey time; the same time frame used to calculate crowdedness). During the data collection period (approximately eight months), 143 security calls were made from the ED occurring within an hour of a completed survey. Importantly, this number of calls is typical for this ED, and for other ED’s in Israel, based on personal communication with the manager of this hospital, and with the manager of the central agency monitoring violent events in Israel's hospitals.

### Psychological measures

Measures of psychological perceptions were collected through self-report surveys distributed by RAs to all people in the ED who agreed to respond.

#### Perceived load

People responded to four items (adapted from [90]) that assessed their perceptions of the level of workload in the ED at the time they responded. A sample item from the survey is: “The ED is very busy right now,” (7-point Likert scale, from 1 = “strongly agree” to 7 = “strongly disagree”; Cronbach's α = 0.77).

#### Perceived future wait

People indicated how long they believed they would still have to wait before completing their ED visit: "How long do you predict that you will continue to wait?”.

### Demographics

We collected people’s age, gender, years of education, socio-economic status, medical reason for ED visit, and number of previous visits to the ED.

### Statistical analyses

We used Hayes’s PROCESS method to analyze our data [91]. All of the Hypotheses (1–4) were examined using PROCESS Model 3 with bootstrapping of 5,000 iterations. PROCESS is a macro for SPSS and is the appropriate statistical method for testing regression analysis of complex relationships between variables, such as moderation or mediation. In this framework the outcome is violence and the predictors are crowdedness, perceived load, and perceived future wait, where past wait acts as a covariate (i.e., a control).

All variables were originally highly skewed and non-normally distributed, with error variances that were not distributed normally. Violence had a skewness of 1.66 (SE = 0.20), crowdedness of 0.61 (SE = 0.19), perceived load of 1.54 (SE = 1.62), perceived future wait of 1.76 (SE = 0.18), and past wait of 1.44 (SE = 0.16). We reduced skewness by following the standard transformation recommendations in such cases [92]. We chose the transformation that produced the highest reduction in skewness for each variable, namely, the square-root transformation resulting in the following skewness values: 0.88 (SE = 0.20) for violence, 0.12 (SE = 0.19) for crowdedness, -0.16 (SE = 0.16) for perceived load, and 0.30 (SE = 0.18) for perceived future wait. The log(e) transformation for past wait resulted in a skewness of 0.30 (SE = 0.16). Results also hold when skewness is reduced only using the square-root transformation.

#### Regression equations by hypotheses

*Y* = Violence
*X*_1_ = Crowdedness
*X*_2_ = Perceived future wait
*X*_3_ = Perceived load

$$\text{H}1:Y\;=\beta_{0}+\beta_{1}X_{1}+e$$

$$\text{H}2:Y\;=\beta_{0}+\beta_{1}X_{1}+\beta_{2}X_{2}+\beta_{4}X_{1}X_{2}+e$$

$$\text{H}3:Y=\beta_{0}+\beta_{1}X_{1}+\beta_{3}X_{3}+\beta_{5}X_{1}X_{3}+e$$

$$\text{H}4:Y=\beta_{0}+\beta_{1}X_{1}+\beta_{2}X_{2}+\beta_{3}X_{3}+\beta_{4}X_{1}X_{2}+\beta_{5}X_{1}X_{3}+\beta_{6}X_{1}X_{2}X_{3}+e$$

### Ethics

The Rambam Medical Center’s Helsinki committee institutional review board and the

Technion—Israel’s Institute of Technology’s ethics committee specifically approved this study.

## Results

Table 1 provides the means, standard deviations, and inter-correlations of all study variables. Participants in the study (*N*) = 226 had been waiting in the ED (95% response rate) anywhere from a few minutes to 12 hours (56% female; *Mean*_*age*_ = 33.99, *SD*_*age*_ = 13.60; *Mean*_*education*_ = 13.31 years, *SD*_*education*_ *=* 2.19 years).

**Table 1:** Means, standard deviations, and inter-correlations between study variables.

		Non-transformed variables		Transformed variables ^a^					
	Unit of Measurement	Mean	SD	Mean	SD	1	2	3	4
1. Past wait	Hours	3.41	2.22	1.04	0.60				
2. Crowdedness	Number of Patients	37.33	22.38	5.82	1.86	0.22**			
3. Perceived future wait	Hours	1.97	1.48	1.30	0.51	0.36**	0.34**		
4. Perceived load	Likert scale 1–7	4.34	2.26	2.01	0.56	0.25**	0.52**	0.37**	
5. Violence	Number of incidents per hour	0.60	0.96	0.46	0.63	-0.01	0.40**	0.11	-0.06

* *p* < 0.05;

** *p* < 0.01 (one-tailed);

Crowdedness, violence, perceived load, and perceived future wait were transformed using square root transformation. Past wait was transformed using log(e) transformation.

The demographic variables did not significantly influence the study variables. Following Carlson and Wu [90], all analyses included the past wait as a control variable, although the pattern and significance of the results did not change when this control variable was excluded from the analyses.

### Hypotheses testing

**Fig 2:**
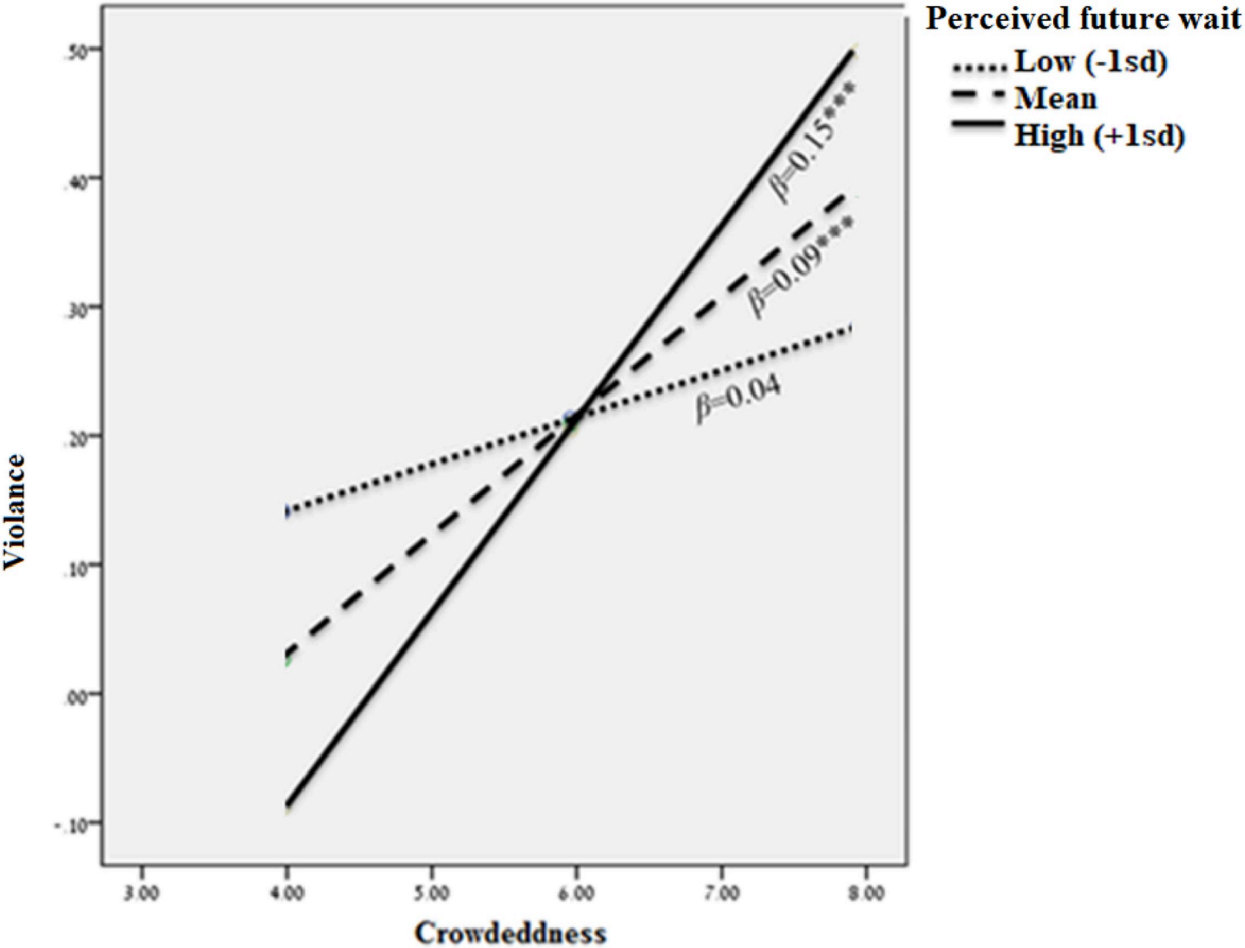
Perceived future wait interacts with crowdedness to predict violence.

Results of hypotheses testing are summarized in Table 2. Hypothesis 1, which predicted that crowdedness is associated with an increased number of violent attacks against service staff, was supported (*β* = 0.1, *p* = 0.004). Hypothesis 2 posited that the perceived future wait buffers the effect of crowdedness on violence, such that the lower the perceived future wait, the weaker the relationship between crowdedness and violence. This hypothesis was also supported (β(crowdedness*perceived future wait) = -0.91, *p* <0.001, 95% CI (-1.43, -0.39)). Indeed, when perceived future wait was low, the effect of crowdedness on violence became non-significant. Moreover, violence was lowest when crowdedness was high and perceived future wait was low, supporting Hypothesis 2 (see Fig 2).

**Table 2:** Testing the full research model for predicting violence.

	Violence *β(SE)*	(95%CI)	
Past wait	-0.06(0.10)	(-0.25, 0.14)	
Crowdedness	0.94**(0.32)	(0.30, 0.58)	
Perceived future wait	4.20*(1.24)	(1.74, 6.65)	
Crowdedness* Perceived future wait	-0.91***(0.26)	(-1.43, -0.39)	
Perceived load	1.48**†** (0.75)	(-0.00, 2.96)	
Crowdedness* Perceived load	-0.39**(0.15)	(-0.68, -0.09)	
Perceived future wait* Perceived load	-2.22***(0.58)	(-3.36, -1.07)	
Crowdedness* Perceived future wait* Perceived load	0.45***(0.11)	(0.22, 0.68)	
R^2^			0.49***

**†***p* < 0.1;

*p < 0.05;

**p <0.01;

***p < 0.001;

boot = 5,000.

**Fig 3:**
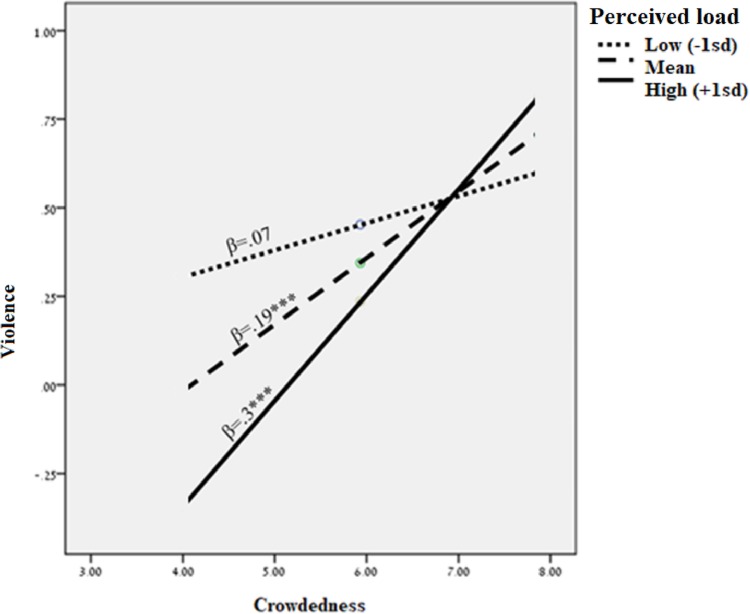
Perceived load interacts with crowdedness to predict violence.

Hypothesis 3 predicted that perceived load buffers the effect of crowdedness on violence, such that the higher the perceived load, the weaker the relationship between crowdedness and violence. This hypothesis was also supported, with a significant interaction of crowdedness and perceived load in their influence on violence (β(crowdedness*perceived load) = -0.39, *p* = 0.01, 95% CI (-0.68, -0.09)). Perceived load buffers the harmful effect of crowdedness on violence such that violence is lowest when the perceived load is high and crowdedness is low, supporting Hypothesis 3 (see Fig 3).

**Fig 4:**
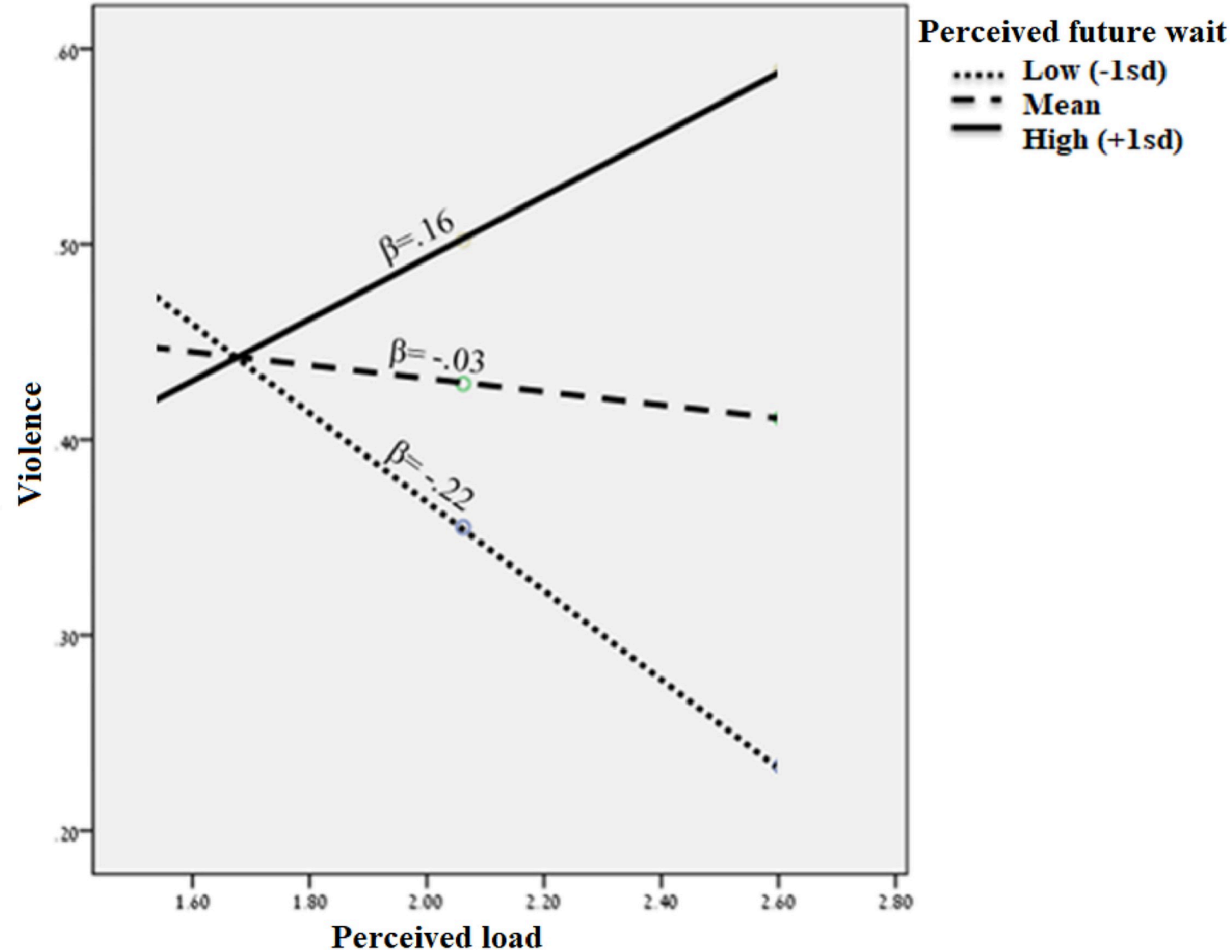
Perceived future wait interacts with perceived load to predict violence.

Hypothesis 4 suggested a three-way interaction between crowdedness, perceived future wait, and perceived load, such that violence is lowest when perceived future wait is lowest and perceived load is highest. This hypothesis was also supported (β(crowdedness*perceived future wait*perceived load) = 0.45, *p* = 0.001, 95% CI (0.22, 0.68); R^2^ = 0.49, *p* <0.001). Results confirm a three-way interaction between crowdedness, perceived future wait, and perceived load in their influence on violence, where crowdedness is associated with higher violence, and high perceived load and low perceived future wait are associated with lower violence (see Fig 4).

## Discussion

Our study shows that the association between crowdedness and violence is moderated by psychological evaluations of future wait and perceived load on the system. These operational variables were impacted by psychological measures of perceived future wait and perceived load. When clients waiting in a crowded ED perceived their future wait time as short, and the load in the ED as high, there was a weaker association between crowdedness and violence. A three-way interaction shows further that high perceived load and low perceived future wait are associated with fewer incidents of violence.

Our results offer a novel way of thinking about the acute problem of violence, especially in cases where space limitations and staff constraints make it difficult to move people more quickly through the system (shortening wait durations) or increase the size of the waiting area (reducing crowdedness). Our findings suggest that showing clients the high workload, together with turning their attention to the future and giving them hope by shortening their perceived future wait (rather than having them focus on the past—the time already lost to waiting) holds promise for reducing violence towards staff.

The behavior of people waiting in queues is the least-understood aspect of queue management [93,94,95]. Our analysis attempts to understand when and why people who are waiting in queues are more (or less) likely to behave violently. To do so we integrate psychological and objective characteristics of queues. We offer theoretical and practical insights into a frequent and harmful outcome associated with queueing—violence towards service providers. As the first step towards reducing aggression and violence we attempt to uncover the underlying process and by in so doing contribute to knowledge regarding aggression in queues, which is both dangerous and costly [41,6,10]. To date, most operational research has examined field data whereas managerial research has relied on survey-based data, and these two research streams have mostly been conducted in isolation. In the present study, we integrate both operational and psychological data to better understand the aspects of queues that are associated with violence.

Specifically, we address two psychological processes that people experience while waiting in a queue. First, we examine the psychological effect of perceived future wait. We demonstrate that when people perceive their remaining wait time as short, this coincides with a decline in rates of violence [77]. Second, we examine the relationship between the perception that the service system is particularly loaded, with the frequency of violence. Previous work suggests that when people perceive a service system as highly loaded, they are more likely to regard the need to wait as justified because of a “labor illusion” [32]. This, in turn, reduces their tendency to act violently towards service providers. We indeed found reduced levels of violence when our respondents perceived the ED to be highly loaded. Moreover, these two psychological assessments interact, such that a shorter perceived future wait appears to be most useful at buffering the effect of crowdedness on violence when the service system is perceived as highly loaded.

### Limitations and future directions

This research project has several limitations that highlight potential directions for future research. First, we examined our predictions using a sample drawn from a hospital ED waiting room and we tested for the relationship between these variables, thus our results do not claim causality. The ED is an extremely stressful environment [96] and, as such, it might not represent all service organizations. However, since violent acts are a low base-rate phenomenon, and it is difficult to obtain empirical data regarding *actual* violence, this setting can serve as an example for other service queues. The present study allowed us to investigate a phenomenon that is rarely examined in the managerial literature using field data. Replicating our results with different samples or with different service organizations characterized by varying degrees of urgency or aggression, may enhance the generalizability of our findings [97].

Second, we measured perceived future wait time using a single-item measure. Despite recent discussion and justification of such means [98], it would be useful for future research to examine the unique construct of perceived future wait and its nomological net more carefully.

Third, we cannot claim that people who responded to the surveys were those who were later violent towards the staff. Moreover, it is important to note that the objective measurement of crowdedness was based on the number of patients in the ED and did not include the number of escorts accompanying them, while the psychological measurement of perceived load included escorts. However, there is no reason to believe that the number of escorts per patient changes over time. Our analysis is at the aggregate level of analysis, of all the people in the ED at a given time, and the total level of aggression at the same time. Because of data privacy issues we could not identify the individuals who responded to the survey, nor the people who were reported as violent. What the surveys provide us, however, is, in aggregate, measures of the psychological climate of the people waiting in the ED, regarding perceived load and perceived future wait. Our analyses show that the aggregate climate measures moderate the extent of the aggregate level of violence in the ED. Future research can hopefully continue this line of thought to examine the individual level relationships.

Fourth, we suggested that short perceived future wait time gives people hope that they will soon attain their goal of receiving service. We were unable to test this suggestion empirically using the current dataset. Yet, hope has strong psychological effects on individuals and it is important for many organizationally relevant variables, such as resilience and self-efficacy [99]. Further research should examine the effect of hope in queues and seek how organizations can spark hope among people who must wait for service.

Fifth, a recent study showed how employees, who are perceived as being distressed as opposed to being passionate, are evaluated less positively by others [94]. These evaluations were made, however, regarding the perceived competency of employees and their chances of being hired, and the study did not investigate service and queue settings. In light of our findings, it might actually be beneficial for employees in busy service settings to show their high workload to others. This still needs to be tested and examined in future research, but it might be that if the service setting is perceived as loaded, service providers will be perceived as working very hard, leading clients to behave less violently towards service staff even if they need to wait for service. As we demonstrated, in a given situation where clients have to wait, they might “appreciate” hard working staff under high perceived load, relative to staff perceived as not working, due to a low workload. Our work is in line with early work on emotional labor and convenience stores [95], which shows that clients were crankier when service providers expressed positive emotions when the store was busy, as it made it seem as if such positivity prolongs the wait and slows the service. However, when the store was not busy, positive emotions were welcomed. Thus, clients have different expectations from service providers when a place is busy, and they appreciate it when service providers seem busy doing “the right things” rather than “wasting time” on things like displaying positive emotions. We call for more work to be done testing this prediction.

Lastly, we must note that there is probably a limit to the effect of low perceived future wait time. If predictions of a short wait are not met, such that the remaining wait is longer than expected, the disillusionment could lead people to become more frustrated and potentially more violent. Thus, lowering people’s perceptions of the remaining wait time may potentially backfire. This effect warrants future investigation.

In addition, we call for future research to focus on practical ways to manipulate these psychological assessments of the objectively crowded service environment that entail long waiting durations, to create the impression of “busyness”, and a perception of “light at the end of the tunnel”.

### Conclusion

We have applied an innovative technique that integrates operational with psychological measures to study the relationship between queues and violence in an ED service setting. Our findings suggest that violence in queues is related to crowdedness and a potential for ameliorating violence is related to psychological assessments. Violence is lowest when people believe they have a short wait a head of them and when they believe the system is busy, an indication that the service providers are working heard. These psychological assessments may have a synergistic effect that can potentially offer a remedy to reduce violence in queues.

## Supporting information

S1 File Original survey English. (DOCX)

S2 File Original survey Hebrew. (DOCX)

S3 File Consent English. (PDF)

S4 File Consent Hebrew. (PDF)

S5 File Scatterplot 1. (TIF)

S6 File Scatterplot 2. (TIF)
