# Correction: How psychology might alleviate violence in queues: Perceived future wait and perceived load moderate violence against service providers

**DOI:** 10.1371/journal.pone.0220395

**Published:** 2019-07-23

**Authors:** Dorit Efrat-Treister, Arik Cheshin, Dana Harari, Shira Agasi, Hadar Moriah, Hanna Admi, Anat Rafaeli

The authors are listed out of order. Please view the correct author order, affiliations, and citation here:

Dorit Efrat-Treister^¶*1^, Arik Cheshin^¶2^, Dana Harari^3^, Shira Agasi^4^, Hadar Moriah^4^, Hanna Admi^5^, Anat Rafaeli^4^

**1** Department of Management, Ben-Gurion University of the Negev, Beersheba, Israel, **2** Department of Human Services, University of Haifa, Haifa, Israel, **3** Scheller College of Business, Georgia Institute of Technology, Atlanta, United States, **4** Faculty of Industrial Engineering and Management, Technion-Israel Institute of Technology, Haifa, Israel, **5** Department of Nursing, The Max Stern Yezreel Valley College, Yezreel Valley, Israel

Efrat-Treister D, Cheshin A, Harari D, Agasi S, Moriah H, Admi H, et al. (2019) How psychology might alleviate violence in queues: Perceived future wait and perceived load moderate violence against service providers. PLoS ONE 14(6): e0218184. https://doi.org/10.1371/journal.pone.0218184

## References

[1] Efrat-Treister D, Cheshin A, Harari D, Rafaeli A, Agasi S, Moriah H, et al (2019) How psychology might alleviate violence in queues: Perceived future wait and perceived load moderate violence against service providers. PLoS ONE 14(6): e0218184 10.1371/journal.pone.0218184 31233514 PMC6590795

