# Supplementary material for: How psychology might alleviate violence in queues: Perceived future wait and perceived load moderate violence against service providers
Source: PLoS One. 2019 Jun 24;14(6):e0218184. doi: 10.1371/journal.pone.0218184 (PMC6590795; doi:10.1371/journal.pone.0218184)
Supplement: S1 File — (DOCX) [file pone.0218184.s001.docx]

Hello and welcome!

We are trying to learn about the the feelings of patients waiting in the Emergency Department. There are no correct or incorrect answers. Your answers will be accessed only by the research team.

1.       What time did you get to the emergency room? ____

2.       For how long have you been waiting? ____

3.       How long do you predict that you will continue to wait? ____

4.       Why did you arrive at the Emergency Department?

Please use the following numbers to indicate your answer to the next few questions:

| **1** | **2** | **3** | **4** | **5** | **6** | **7** |
| --- | --- | --- | --- | --- | --- | --- |
| Strongly Disagree | Disagree | Slightly Disagree | Neutral | Slightly Agree | Agree | Strongly Agree |

To what degree do you agree with the following statements? (from 1 to 7)

5.       The Emergency Department is very busy right now _______

6.       There are many people in the Emergency Department right now _______

7.       The Emergency Department is very crowded right now _______

8.       I feel that there are many people here _______

For the study's purposes, we ask you to provide us with some personal details. We would appreciate it if you'd share a few details with us.

All of your answers are confidential, and your anonymity is guaranteed.

9.       Age: _______

10.       Gender: Male/Female

11.       Years of education _______

Thank you for your assistance!
