# Supplementary material for: How psychology might alleviate violence in queues: Perceived future wait and perceived load moderate violence against service providers
Source: PLoS One. 2019 Jun 24;14(6):e0218184. doi: 10.1371/journal.pone.0218184 (PMC6590795; doi:10.1371/journal.pone.0218184)
Supplement: S2 File — (DOCX) [file pone.0218184.s002.docx]

שלום רב!

אנו מנסים ללמוד על תחושות של מטופלים הממתינים במחלקה לרפואה דחופה. אין תשובות נכונות או לא נכונות. התשובות יועברו רק לצוות החוקרים.

| 1.    באיזו שעה הגעת לחדר המיון?  2.    כמה זמן את/ה כבר ממתין?  3.        כמה זמן אתה מעריך שעוד תמתין?  4.        למה הגעת למיון? |  |
| --- | --- |

אנא השתמש במספרים הבאים לציון תשובתך על השאלות הבאות:

| 7 =  במידה  רבה מאוד | 6 =  במידה  רבה | 5 =  במידה  די רבה | 4 =  במידה בינונית | 3 =  במידה  די מועטה | 2 =  במידה מועטה | 1 =  במידה מועטה מאוד |
| --- | --- | --- | --- | --- | --- | --- |

עד כמה את/ה מסכים/מה עם כל אחת מהאמירות הבאות? (מ-1 עד 7)

5.   חדר המיון עמוס כרגע: ________

6.   יש עכשיו המון אנשים בחדר המיון: ________

7.   מאד צפוף במיון כרגע: ________

8.   אני מרגיש שיש פה הרבה אנשים: ________

לצרכי המחקר אנו מבקשים ממך למלא מספר פרטים אישיים. כל תשובותיך הן אנונימיות ותשמרנה בחיסיון מוחלט.

| 9.   גיל: ______ |  |
| --- | --- |
| 10.   מגדר: זכר/ נקבה:_______ |  |
| 11.   מספר שנות לימוד:_______ |  |

תודה רבה על עזרתך!
