# Supplementary material for: How psychology might alleviate violence in queues: Perceived future wait and perceived load moderate violence against service providers
Source: PLoS One. 2019 Jun 24;14(6):e0218184. doi: 10.1371/journal.pone.0218184 (PMC6590795; doi:10.1371/journal.pone.0218184)
Supplement: S3 File — (PDF) [file pone.0218184.s003.pdf]

Form #2

Informed consent to participate in a medical study that is not a medical experiment on human beings.

Helsinki committee request number: **0216-09-RMB**

I, as identified below:

First and last name:	
ID number:	
Address:	Zip code:

A) **Declare that I Agree to participate in the research, as detailed in this form.**

B) **Declare that the following clauses were explained to me by:**

Name of the explaining researcher\research assistant:

(1) **The leading researcher** Dr. Yael Aylon **was granted permission to perform the research by the medical establishment's director**

(2) **The study is about:**

Anger, aggression, and violence in hospitals – identifying the circumstances that lead to the phenomenon, it's implications and recommendations to lessen it.

(3) **I am free to choose not to participate in this study, and I am free to stop participating at any time, all without harming my right to receive treatment.**

(4) **My identity will be kept confidential by all involved in the research, and it will not be published anywhere, including scientific journals.**

(5) **In the case of filling the questionnaire – I am permitted to not answer all of the questions, or some of them.**

C) **I declare that I was given detailed information about the study, especially about the following details:**

(1) **Goals**

Identifying the circumstances and the process that leads to aggression and violence in hospitals, recognizing the consequences, and recommending ways of preventing aggression and violence in hospitals.

(2) **As part of the study, the participant is required to:**

1. **Filling the questionnaire**
2. **Participate in a personal interview**

(3) The inconvenience that may be caused

There should not be any inconvenience**.**

D) I declare that I have given my consent out of my own free will, and I have understood all what is written above.

Additionally, I have received a copy of the informed consent form and the attachment (if there is one).

I am aware that if I have questions regarding this study, I can contact Dr. Yael Aylon **via phone\answering machine:** 04-8543116, 050-2062368

Participant's Name:	Participant's Signature:	Date:

Researcher's\Research Assistant's Declaration:

Name of the explaining researcher\research assistant:	Signature:	Date:
