# Supplementary material for: How psychology might alleviate violence in queues: Perceived future wait and perceived load moderate violence against service providers
Source: PLoS One. 2019 Jun 24;14(6):e0218184. doi: 10.1371/journal.pone.0218184 (PMC6590795; doi:10.1371/journal.pone.0218184)
Supplement: S4 File — (PDF) [file pone.0218184.s004.pdf]

טופס 2

הסכמה מדעת להשתתפות במחקר שאינו ניסוי רפואי בבני אדם

מספר הבקשה בוועדת הלסינקי :
0216-09 -RMB

אני החתום מטה:

שם פרטי ומשפחה:	
מס' תעודת זהות:	
כתובת:	מיקוד:

א) מצהיר/ה בזה כי אני מסכים/ה להשתתף במחקר כמפורט במסמך זה.

ב) מצהיר/ה בזה כי הוסבר לי על- ידי:

שם החוקר/חוקר המשנה המסביר:

(1) כי החוקר הראשי ד"ר יעל אילון **קיבל ממנהל המוסד הרפואי אישור לביצוע המחקר.**

(2) כי המחקר נערך בנושא:
כעס, אגרסיביות ואלימות בבית החולים - זיהוי הנסיבות המובילות לתופעה, השלכותיה והמלצות להפחתתה.

(3) כי אני חופשי/ה לבחור שלא להשתתף במחקר, וכי אני חופשי/ה להפסיק בכל עת השתתפותי במחקר, כל זאת מבלי לפגוע בזכותי לקבל את הטיפול המקובל.

(4) כי מובטח שזהותי האישית תשמר סודית על ידי כל העוסקים והמעורבים במחקר ולא תפורסם בכל פרסום כולל בפרסומים מדעיים.

(5) כי במקרה של מילוי שאלון – אני רשאי/ת שלא לענות על כל השאלות שבשאלון או על חלק מהן.

ג) הנני מצהיר/ה כי נמסר לי מידע מפורט על המחקר ובמיוחד על הפרטים הבאים המפורטים להלן/המפורטים בדף מידע המצורף לטופס זה[1]:

(1) מטרות
זיהוי הנסיבות והתהליכים המובילים לאגרסיביות ואלימות בבית החולים, זיהוי ההשלכות, מתן המלצות ראשוניות למניעת אגרסיביות ואלימות בבית החולים

(2) הנדרש מהמשתתף במסגרת המחקר
1. מילוי שאלון
2.□ השתתפות בראיון אישי

(3) אי-הנוחות העלולה להיגרם
לא צפויה אי נוחות כלשהי

ד) הנני מצהיר/ה בזה כי הסכמתי הנ"ל נתתי מרצוני החופשי וכי הבינותי את כל האמור לעיל. כמו כן קיבלתי עותק של טופס ההסכמה מדעת ואת דף המידע המצורף לטופס זה (אם קיים).

ידוע לי כי בכל שאלה הקשורה למחקר אוכל לפנות ל- ד"ר יעל אילון **מספר טלפון/משיבון:** 04-8543116, 050-2062368

שם המשתתף/ת במחקר	חתימת המשתתף/ת במחקר	תאריך

הצהרת החוקר/חוקר המשנה:

שם החוקר/חוקר המשנה שהסביר:	חתימתו	תאריך

את המידע בסעיף ג' ניתן לפרט בדף מידע נפרד שיצורף לטופס זה

End of document
