# Supplementary figures and images for: How psychology might alleviate violence in queues: Perceived future wait and perceived load moderate violence against service providers
Source: PLoS One. 2019 Jun 24;14(6):e0218184. doi: 10.1371/journal.pone.0218184 (PMC6590795; doi:10.1371/journal.pone.0218184)

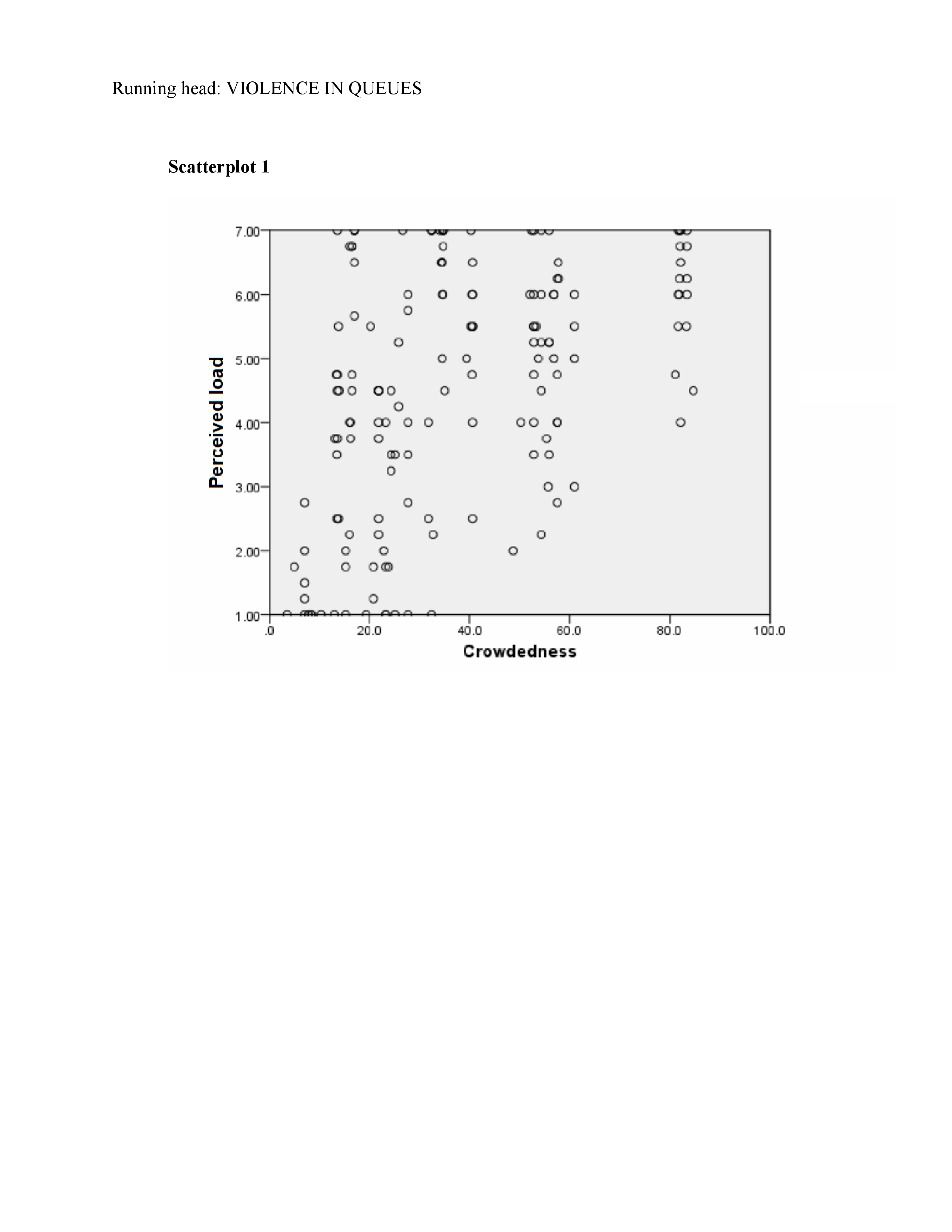

Supplement: S5 File — (TIF) [file pone.0218184.s005.tif]

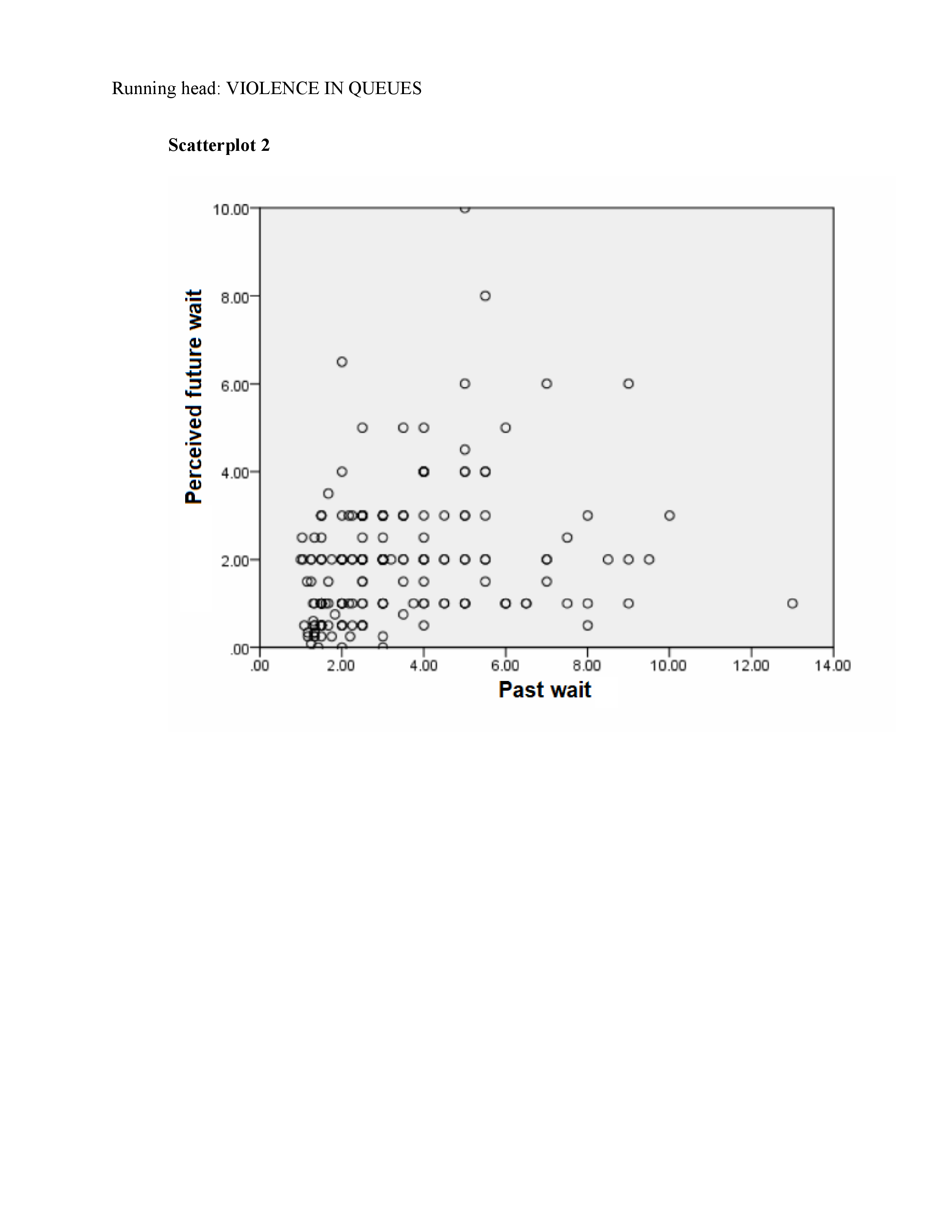

Supplement: S6 File — (TIF) [file pone.0218184.s006.tif]
